# 

**Published:** 1914-08-08

**Authors:** 


### Medical Appointments.

Mr. Claude Harry Mills, M.R.C.S., L.R.C.P., has been appointed an honorary assistant surgeon to St. Paul's Hospital for Skin and Genito-Urinary Diseases, Red Lion Square, Holborn, W.C.

Dr. A. A. W. Petrie, third assistant medical officer at Hanwell Mental Hospital, has been appointed to be assistant medical officer at the London County Council's Epileptic Colony, on the understanding that his services may be utilised for general purposes connected with the Lunacy and Mental Deficiency Services for the County of London, as well as for work at the Colony.

Rates of Subscription: Twelve months, 6/6; Six months, 4/-; Three months, 2/-, post free to any address in the United Kingdom. Abroad, Twelve months, 8/8; Six months, 5/-; Three months, 2/6, post free.—Address The Subscription Dept., "The Hospital," The Hospital Building, 28/29 Southampton Street Strand, London W.C.

